# Activation of TSLP–IL-9 Axis Hinders the Antifibrotic Effect of ST2 Deficiency in Pulmonary Fibrosis

**DOI:** 10.3390/ijms262411787

**Published:** 2025-12-05

**Authors:** Sergei P. Atamas, Virginia Lockatell, Zhongcheng Mei, Mohan E. Tulapurkar, Katerina N. Lugkey, Alexander Sasha Krupnick, Irina G. Luzina

**Affiliations:** 1 Department of Medicine, University of Maryland School of Medicine, Baltimore, MD 21201, USA; 2 Department of Surgery, University of Maryland School of Medicine, Baltimore, MD 21201, USA; 3 Research Service, Baltimore VA Medical Center, Baltimore, MD 21201, USA

**Keywords:** cytokines, fibrosis, lung, animal models

## Abstract

Previous studies have suggested that activation of the IL-33/ST2 axis as well as elevated expression of the full-length IL-33 precursor acting in an ST2-independent fashion both contribute to pulmonary fibrosis. The protective effect of genetic ST2 deficiency on pulmonary fibrosis is known to be partial, with unclear mechanisms preventing a more complete protection. Here, we report that ST2 deficiency failed to fully protect the lungs from excess collagen accumulation after the profibrotic bleomycin injury and simultaneously facilitated elevations in pulmonary levels of a previously suggested profibrotic mediator, IL-9, as well as a known activator of IL-9 expression, TSLP. Pulmonary CD4+ T cells were the main producers of IL-9. Neutralizing antibody-mediated in vivo blockade of TSLP potently attenuated pulmonary levels of both IL-9 and collagen in the bleomycin injury model in wild-type and particularly ST2-deficient mice. All these observations were markedly pronounced in mice with single deficiency of ST2 and the overall pattern of findings was also preserved in mice with dual deficiency of ST2 and IL-33. It was concluded that the antifibrotic effect of ST2 deficiency is hindered by the simultaneous activation of the TSLP–IL-9 axis in experimental bleomycin-induced pulmonary fibrosis. These findings inform further development of antifibrotic therapies.

## 1. Introduction

**Figure 1:**
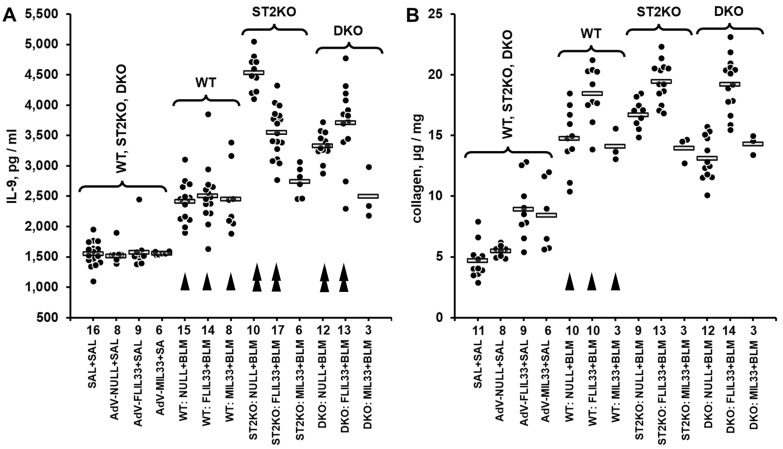
Pulmonary levels of IL-9 measured by ELISA (**A**) or collagen measured based hydroxyproline content (**B**) in lung homogenates from mice challenged with intratracheal bleomycin injury in combination with either FLIL33 or MIL33 overexpression. In both panels, dots indicate the measured levels in pulmonary homogenates of individual mice adjusted for the dilution factor, whereas horizontal bars indicate mean values for each of the experimental groups. Selected pairwise significant (*p* < 0.05) differences are indicated by arrowheads. In both panels, single arrowheads indicate differences in WT bleomycin-treated mice compared to the corresponding saline-treated controls, whereas double arrowheads indicate differences in bleomycin-treated genetically manipulated mice compared to the corresponding bleomycin-treated WT animals. (**A**) In this combined dataset from a series of double-hit experiments, animal groups are marked based on their genetic background (WT, ST2KO, DKO), indication of the IL-33 form delivered in the first hit by intratracheal instillation of AdV constructs (FLIL33, MIL33, or NULL control), and indication of the second hit (SAL for saline or BLM for bleomycin). The numbers of tested animals for each experimental group are indicated immediately below the horizontal axis. In the control group on the left (SAL + SAL), approximately equal numbers of animals of each strain were tested, revealing no significant differences in the basal level of IL-9; these data were combined into the single group of 27 animals for clarity of data presentation. Additionally, to assess the effects of AdV-based gene delivery alone, approximately equal numbers of animals of each genetic background received either AdV-FLIL33, AdV-MIL33, or AdV-NULL, followed by the second hit with saline, and then tested for pulmonary levels of IL-9. The ANOVA test revealed significant (*p* < 0.001) differences across all 13 tested groups of animals. (**B**) Pulmonary levels of collagen in a subset of mice tested for IL-9 as indicated in (**A**). The ANOVA test revealed significant (*p* < 0.001) differences across the tested groups of animals.

The pathophysiology of pulmonary fibrosis in general, including the complexity of the pro-and antifibrotic regulation by immune mechanisms, remains far from fully understood [1,2,3,4,5,6]. Such incomplete understanding makes pulmonary fibrosis a difficult-to-treat health condition with no cure. Interleukin (IL)-33 and its cell-surface receptor, interleukin-1 receptor-like 1 (IL1RL1), also known as T1/ST2 or simply ST2, have emerged as potentially therapeutically targetable mediators of the fibrotic process in the lungs [7,8,9,10,11,12,13,14,15]. In this report, we use the common designation of the IL-33’s receptor as ST2, referring to “serum stimulation-2” but not “suppressor of tumorigenicity 2.” The latter name has been often incorrectly used in literature in relevance to the IL-33 pathway, as others have pointed out [16,17,18,19]. Homozygous genetic deletion, or knockout (KO), of either IL-33 (IL33KO), ST2 (ST2KO), or both (IL33KOST2KO, or double-knockout [DKO]) attenuates the magnitude of bleomycin-induced pulmonary fibrosis [8,9,13]. However, IL-33 and ST2 may regulate fibrosis not only through the conventionally considered IL-33/ST2 ligand/receptor axis, but also independently of each other, with each IL-33 and ST2 acting through distinct pathways. Indeed, not only the proteolytically mature IL-33 (MIL33) cytokine acting through ST2 but also the full-length IL-33 (FLIL33) precursor in the complete absence of ST2 promotes pulmonary fibrosis [8,9,10,11,20]. Interestingly, genetic deletion of IL-33 appears to attenuate bleomycin-induced accumulation of pulmonary collagen in mice more potently than does genetic deletion of ST2 (compare Figure 1D and Figure 3A in [9]). The current study sought to begin exploration of possible mechanisms that potentially maintain such residual fibrotic response, particularly in ST2-deficient mice.

Interleukin-9, the signature cytokine of the Th9 immune response which plays important roles in allergic, autoimmune, and oncological pathologies [21,22,23,24], has also been implicated as a regulator of lung fibrosis. In part functionally overlapping with the Th2 pathway [25,26], the cellular sources of IL-9 produce this cytokine in response to IL-33-induced activation through ST2. The binding of IL-33 to ST2 leads to IL-9 production by T cells, basophils, mast cells, and by ILC2 in the lung and other tissues in health and disease [21,22,23,24,25,26,27,28,29,30,31,32,33,34]. Production of IL-9 can be induced by other factors, e.g., thymic stromal lymphopoietin (TSLP) [35,36] or transforming growth factor (TGF)-β [37,38], even in the absence of the activated IL-33/ST2 axis. For example, in a model of allergen-induced experimental asthma in ST2KO mice, deletion of ST2 led to elevated TSLP production which in turn stimulated the emergence of IL-9-producing ILC2 cells [36]. Expression of IL-9 is also regulated epigenetically in a complex fashion [39]. The fact that the levels of IL-9 are elevated in human patients with ILD is well established [40,41,42,43,44], although a controversy remains as to whether such elevations drive a profibrotic regulation or represent failed attempts at an anti-fibrotic control. Suggestive of the profibrotic mechanism, Jiang et al. [43] reported that in their study of patients with connective tissue diseases including SSc, increased serum levels of IL-9 were associated with higher frequency and severity of pulmonary fibrosis. Also pointing to the profibrotic effect of elevated IL-9, Lim at al. [44] observed a strong correlative link between elevated IL-9 mRNA and modified Rodnan skin score, which is an indicator of disease progression in SSc. By contrast, supportive of the protective antifibrotic effect of IL-9, Yanaba et al. [42] reported that elevated systemic levels of IL-9 in patients with SSc were associated with lower frequency and severity of pulmonary fibrosis. In light of the controversial implications of such associative data, more direct, mechanistic studies are necessary to assist with understanding the role of IL-9 in fibrotic regulation. Unfortunately, despite more than two decades of experimental studies in animals, no definitive consensus on this topic has been reached. In a series of articles [45,46,47], Arras et al. reported that overexpression of IL-9 in a transgenic mouse strain was protective in both silica- and bleomycin-induced models of lung fibrosis. However, in the same IL-9-overexpressing transgenic mouse strain, IL-9 played a profibrotic role in airway remodeling in a model of asthma [48]. Furthermore, in the silica model of lung fibrosis in wild-type (in contrast to the IL-9-overexpressing transgenic) mice, neutralization of IL-9 by a blocking antibody suppressed both pulmonary inflammation and fibrosis [40]. In the latter study, IL-9 was produced mostly by pulmonary macrophages and T cells. Moreover, in their exhaustive study [49], Deng et al. investigated the involvement of Th9 cells in pulmonary fibrosis. Among numerous findings in their data-rich report, the authors discovered that Th9 cells are present in increased numbers in the lungs of patients with IPF and mice challenged with bleomycin. Furthermore, administration of an anti-IL-9 neutralizing antibody attenuated the number of pulmonary Th9 cells as well as severity of pulmonary fibrosis.

In this report, we describe and reflect on a newly discovered link between ST2 deficiency and TSLP-driven IL-9 elevation in the regulation of pulmonary fibrosis. This link has important implications for the development of future antifibrotic therapies.

## 2. Results

### 2.1. ST2 Deficiency Potentiates the Double-Hit Injury-Induced Elevation in Pulmonary IL-9 While Failing to Attenuate Pulmonary Fibrosis

To begin exploration of possible mechanisms maintaining the residual fibrotic response in mice deficient of ST2, we screened the levels of pulmonary cytokines in a double-hit model of pulmonary fibrosis in ST2KO and WT mice. Double-hit models are generally considered more relevant to human diseases than single-hit models. We have previously described an in vivo model in which recombinant replication-deficient adenovirus (AdV)-mediated overexpression of either FLIL33 or MIL33 (first hit) is followed by intratracheal bleomycin challenge (second hit) to induce fibrotic changes in the lungs of mice [8,9,10] (Supplementary Figure S1A). The protein levels of a panel of 14 pulmonary cytokines were screened in lung homogenates utilizing the Luminex-based multiplex approach. Among the screened cytokines, there were notable findings related to IL-9, the injury-induced pulmonary levels of which were not attenuated in ST2KO mice (Supplementary Dataset S1 and Figure S1B). Considering the screening nature of the Luminex testing, the more conventional ELISA approach was used to corroborate this observation. Additional double-hit experiments (Supplementary Figure S1A) were performed on seven independent occasions. The levels of IL-9 were measured by ELISA in lung homogenates, and the data from these seven experiments were pooled together as shown in Figure 1A. In these experiments, in addition to animals challenged with bleomycin as the second hit as shown in Supplementary Figure S1B, a separate group of animals received intratracheal saline as the second hit on day 7 post AdV instillation (Figure 1A). One-way ANOVA revealed significant differences in IL-9 expression levels across the experimental groups (*p* < 0.001); statistical significance of pairwise comparisons between the experimental groups is indicated in Supplementary Dataset S2.

In parallel, the levels of pulmonary collagen were measured in a subset of these lung homogenate samples (Figure 1B). One-way ANOVA revealed significant differences in pulmonary collagen levels across the experimental groups (*p* < 0.001); statistical significance of pairwise comparisons between the experimental groups is indicated in Supplementary Dataset S2. Of note, overexpression of neither mature IL-33 cytokine nor full-length IL-33 precursor further elevated pulmonary IL-9 in a notable fashion compared to AdV-NULL control. Moreover, despite some numerical differences between the matching experimental groups of ST2KO and DKO mice, they manifested an overall similar pattern of higher expression of IL-9 compared with WT mice (Figure 1A). Thus, it appeared that the deficiency of ST2 rather than regulation by IL-33 had a more pronounced effect on the induction of IL-9 in the lungs in response to the dual injury. Therefore, subsequent experiments tested whether a similar ST2 deficiency-dependent regulation of pulmonary cytokines may take place in a less complex, single-hit, acute bleomycin injury model.

### 2.2. Bleomycin Injury-Induced Elevation in Pulmonary IL-9 Persists and Is Enhanced Is ST2-Deficient Mice

**Figure 2:**
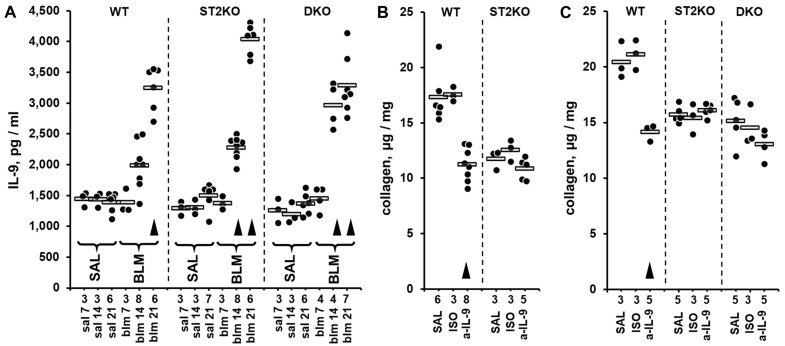
Pulmonary levels of IL-9 measured by ELISA (**A**) or collagen measured based hydroxyproline content (**B**,**C**) in lung homogenates from mice challenged with intratracheal bleomycin injury without (**A**) or with (**B**) treatments with anti-IL-9 blocking antibody. In all panels, dots indicate the measured levels in pulmonary homogenates of individual mice adjusted for the dilution factor, whereas horizontal bars indicate mean values for each of the experimental groups. The numbers of tested animals for each experimental group are indicated immediately below the horizontal axes. Selected pairwise significant (*p* < 0.05) differences are indicated by arrowheads. In each panel, for each indicated strain of mice, arrowheads indicate differences from the corresponding saline-treated controls. (**A**) In this combined dataset from a series of single-hit experiments, animal groups are marked based on their genetic background (WT, ST2KO, DKO), indication of bleomycin (BLM) or saline control (SAL) challenge, and timing of the experimental readout (7, 14, or 21 days post bleomycin injury). The ANOVA test revealed significant (*p* < 0.001) differences across all 13 tested groups of animals. (**B**) The effects of in vivo IL-9 blockade with the anti-IL-9 neutralizing antibody (a-IL-9), compared with saline (SAL) and isotype control antibody (ISO), on pulmonary levels of collagen in WT and ST2KO mice on day 14 post bleomycin injury. The ANOVA test revealed significant (*p* < 0.001) differences across the tested groups of animals. (**C**) The effects of in vivo IL-9 blockade with the anti-IL-9 neutralizing antibody (a-IL-9), compared with saline (SAL) and isotype control antibody (ISO), on pulmonary levels of collagen in WT and ST2KO mice on day 21 post bleomycin injury. The ANOVA test revealed significant (*p* = 0.001) differences across the tested groups of animals.

In the single-hit injury experiment, ST2KO and DKO mice were challenged, along with WT control mice, with a single IT dose of either bleomycin solution or saline control. The animals were euthanized on days 7, 14, or 21, and the protein levels of a panel of 43 pulmonary cytokines were screened in lung homogenates utilizing the proximity extension assay-based Olink multiplex approach. The diagram of experimental design for this Olink-based screening experiment is shown in Supplementary Figure S2A. The Olink Target 48 Mouse screening of 43 biomarkers was performed mostly in the lung homogenates from bleomycin-challenged mice. In addition to samples from bleomycin-challenged mice, lung homogenates from one saline-challenged, day 14, animal in each WT, ST2KO, and DKO groups were also screened for cytokine levels and combined into the saline control group. The overall findings in the Olink-based screening assay were unremarkable for the majority of the 43 tested biomarkers, except for IL-9 (Supplementary Dataset S3 and Figure S2B). The pulmonary levels of IL-9 were notably elevated on days 14 and 21 after the bleomycin challenge, including in mice with germline deficiencies of either ST2 or both IL-33 and ST2 (Supplementary Dataset S3 and Figure S2B). Considering the screening nature of Olink testing, the more conventional ELISA approach was used to corroborate these observations. The levels of IL-9 in lung homogenates from all bleomycin-challenged animals that were used for the Olink-based screening, and, additionally, in lung homogenates from all saline-challenged controls (Supplementary Figure S2A) were measured by ELISA. Additionally, two independent bleomycin injury experiments were performed and the data on pulmonary levels of IL-9 measured by ELISA were pooled with the ELISA data from the samples used for the Olink screening. The combined dataset is presented in Figure 2A. Such ELISA testing confirmed elevated IL-9 levels on days 14 and 21 across the tested mouse strains (Figure 2A). One-way ANOVA revealed significant differences in IL-9 expression levels across the experimental groups (*p* < 0.001); statistical significance of pairwise comparisons between the experimental groups is indicated in Supplementary Dataset S4. Although the overall levels of pulmonary IL-9 in the single-hit bleomycin model were lower than those observed in the double-hit model, the elevations in IL-9 followed the same pattern, with ST2 deficiency causing greater increases. On day 14 after bleomycin injury (paralleling the 14 days post bleomycin exposure in the double-hit model), the increase in pulmonary IL-9 in WT mice did not reach statistical significance whereas the corresponding increase in ST2KO and DKO mice was significant (Supplementary Dataset S4). The elevations were particularly overt on day 21, at which the levels of pulmonary IL-9 were the highest in all three mouse strains, with the differences between the strains not reaching the levels of statistical significance (*p* > 0.05).

To determine whether elevated IL-9 contributes to pulmonary fibrosis, including in the absence of ST2, in vivo neutralization experiments with anti-IL-9 blocking antibodies were performed (Figure 2B,C). On both day 14 (Figure 2B, Supplementary Dataset S5) and day 21 (Figure 2C, Supplementary Dataset S6) after bleomycin injury, the levels of pulmonary collagen in WT mice were moderately attenuated by the treatment with anti-IL-9 blocking antibody but not the corresponding isotype control antibody. This observation suggested that IL-9 contributes to the profibrotic effect of bleomycin in WT mice. As expected based on previous reports [8,9,13], the pulmonary levels of collagen were overall lower in ST2KO and DKO mice, yet the in vivo IL-9 blockade with the neutralizing antibody failed to attenuate these levels further at either 14 (Figure 2B, Supplementary Dataset S5) or 21 days in either ST2KO or DKO mice (Supplementary Dataset S6). The possibility was considered that the potent increase in the levels of IL-9 in ST2-deficient mice could not have been overcome by the used dosing regimen of the blocking antibody.

### 2.3. TSLP Controls Elevations in Pulmonary IL-9 and Collagen, Including in ST2-Deficient Mice

Similarly to our findings in the bleomycin model, Verma et al. [36] observed that in a murine experimental model of asthma, both IL-9 and TSLP were elevated in the lungs of allergen-challenged mice with ST2 deficiency. Furthermore, they observed that administration of a blocking anti-TSLP antibody attenuated induction of IL-9. We therefore tested whether TSLP levels were elevated in the lungs of mice challenged with bleomycin, and whether antibody-mediated blockade of TSLP attenuates IL-9 levels in the bleomycin model.

**Figure 3:**
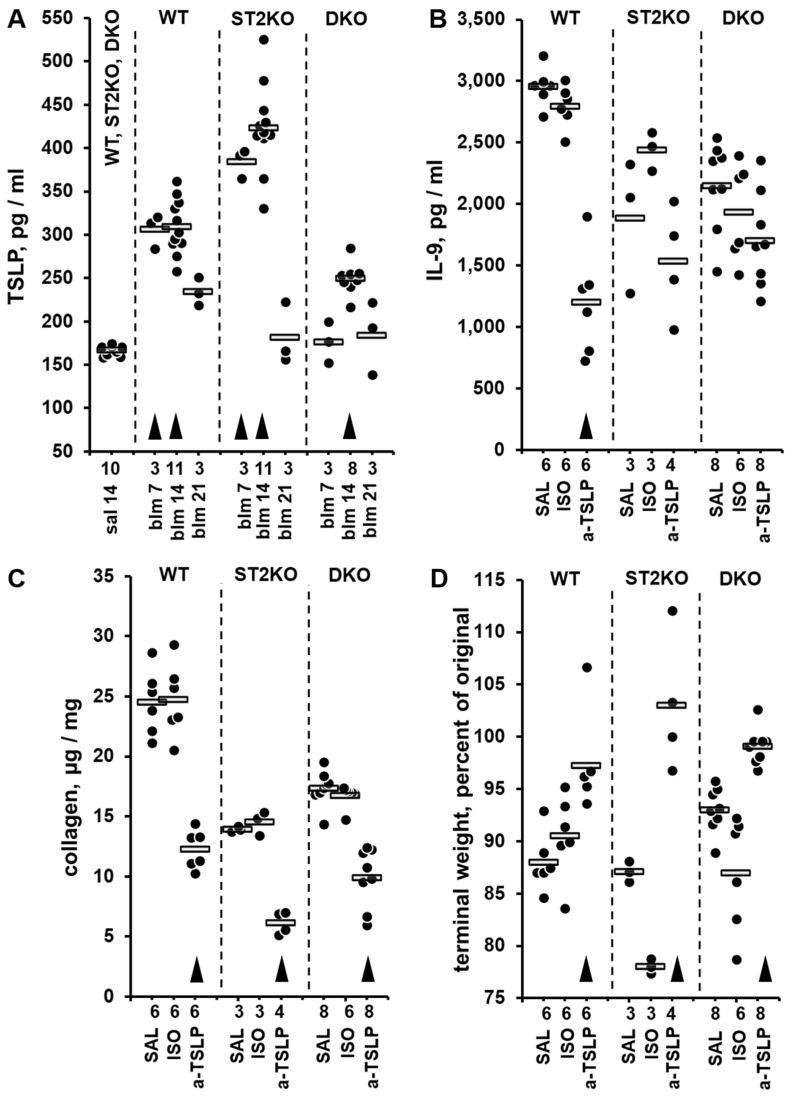
Pulmonary levels of TSLP measured by ELISA in lung homogenates from mice in the bleomycin injury model (**A**) and the effects of in vivo blockade of TSLP with a neutralizing antibody (**B**–**D**). In all panels, dots indicate the measured levels in pulmonary homogenates of individual mice adjusted for the dilution factor, whereas horizontal bars indicate mean values for each of the experimental groups. The numbers of tested animals for each experimental group are indicated immediately below the horizontal axes. For each of the panels, the ANOVA test revealed significant (*p* < 0.001) differences across the tested groups of animals. (**A**) In this combined dataset from three single-hit experiments, animal groups are marked based on their genetic background (WT, ST2KO, DKO), indication of bleomycin (BLM) or saline control (SAL) challenge, and timing of the experimental readout (7, 14, or 21 days post bleomycin injury). In the combined SAL group, approximately equal numbers of animals of each strain were tested, revealing no significant differences in the basal levels of TSLP; these data were combined into the single group of 10 animals for clarity of data presentation. Arrowheads indicate significant (*p* < 0.05) differences from the combined SAL group. (**B**–**D**) Pulmonary levels of IL-9 (**B**) and collagen (**C**) as well as terminal body weight, percent of the initial value before the intratracheal bleomycin challenge (**D**) in animals treated with anti-TSLP (a-TSLP) or isotype control (ISO) antibodies or saline (SAL) control. All readouts were performed on day 21 after bleomycin challenge. In panels (**B**–**D**), for each indicated strain of mice, arrowheads indicate differences from the corresponding saline-treated controls.

Three single-hit, intratracheal bleomycin injury experiments were performed on independent occasions, and the levels of TSLP were measured in lung homogenates by ELISA (Figure 3A). One-way ANOVA revealed significant differences in TSLP expression levels across the experimental groups (*p* < 0.001); statistical significance of pairwise comparisons between the experimental groups is indicated in Supplementary Dataset S7. Notably, pulmonary levels of TSLP were elevated as early as day 7 after the bleomycin challenge in WT and ST2KO mice, and peaked on day 14 across all tested strains, declining substantially on day 21 (Figure 3A, Supplementary Dataset S7). To definitively determine whether TSLP controls pulmonary levels of IL-9 and, consequently, the degree of pulmonary fibrosis, a neutralizing anti-TSLP antibody was used to block the functional effects of TSLP in the single-hit bleomycin model on day 21 after bleomycin challenge (Figure 3B–D). Such blockade of TSLP strongly downregulated pulmonary levels of IL-9 (Figure 3B) and collagen indicative of attenuation of pulmonary fibrosis (Figure 3C) as well as upregulated terminal body weight indicative of alleviation of the overall disease severity (Figure 3D). One-way ANOVA revealed significant differences across the experimental groups (*p* < 0.001) for each of these three variables; statistical significance of pairwise comparisons between the experimental groups for each of the variables is indicated in Supplementary Dataset S8. It was concluded that ST2 deficiency leads to elevated pulmonary IL-9 levels and resistance to IL-9 inhibition through a TSLP-dependent mechanism.

### 2.4. Pulmonary CD4+ T Cells Are the Main Producers of IL-9 in the Bleomycin Injury Model in Both WT and ST2KO Mice

**Figure 4:**
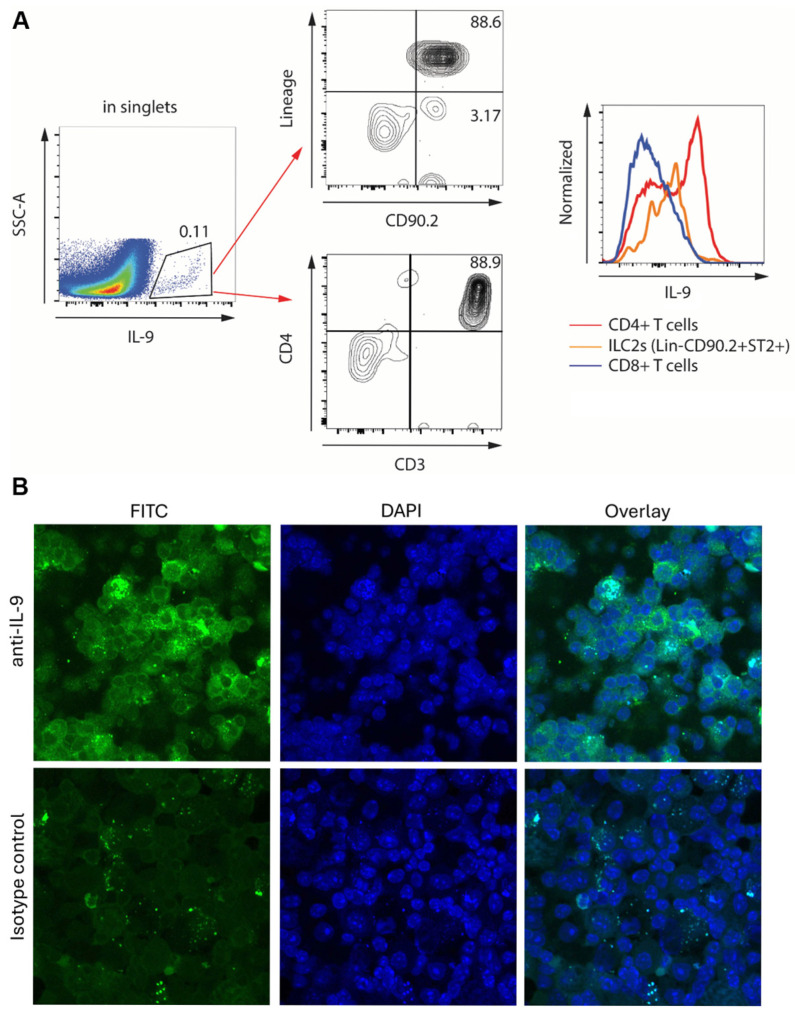
Expression of IL-9 in unseparated lung cells (**A**) or purified T cells (**B**) from bleomycin-treated mice. Similar observations were made in cells from both WT and ST2KO mice. (**A**) Flowcytometric analysis of unseparated lung cells. The IL-9-expressing population (left) was analyzed for cell surface markers characteristic of innate lymphoid cells (CD90.2^+^Lin^−^) as well as for T cell markers CD3 and CD4. Histograms on the right demonstrate relative expression of IL-9 by the indicated cell populations based on fluorescence intensity. (**B**) Confocal microscopy of purified pulmonary T cells stained with FITC-labeled anti-IL-9 or isotype control antibodies (left) or DAPI to reveal nuclei (middle). The overlay of the two fluorescent channels is shown on the right.

Although CD4+ T lymphocytes are thought to be the main producers of IL-9—making them the Th9 cells—numerous cell types are known to be able to produce IL-9 [21,22,23,24]. In the lungs, such major IL-9 producers in addition to Th9 cells can be group 2 innate lymphoid cells, macrophages, and epithelial cells [40,50,51]. To determine the cellular source of IL-9 in our model, flow cytometric analyses of unseparated lung cells were performed (Figure 4A). Gating on the IL-9-positive population of cells, analysis of cell surface markers revealed that the majority of IL-9 producers were CD4+ T cells, while some ILC2 also produced this cytokine. Based on fluorescence intensity, CD4+ T cells expressed higher levels of IL-9 per cell (Figure 4A). To confirm these findings using an independent technique, immunocytochemical staining of purified pulmonary T cells was performed (Figure 4B). The cells stained brightly for intracellular IL-9, whereas similar staining with an isotype control antibody was notably less pronounced. Both flowcytometric and immunohistochemical analyses of lung cells from WT and ST2KO mice similarly demonstrated the predominant production of IL-9 by CD4+ T cells. It was concluded that Th9 cells were the main producers of pulmonary IL-9 in the presence or absence of ST2.

## 3. Discussion

The findings of this study offer an explanation for the previously observed discrepancy between the pronounced antifibrotic effect of IL-33 deficiency and the relatively modest antifibrotic effect of the IL-33-specific receptor, ST2, deficiency [9]. The data presented here indicate that in the absence of ST2, the pulmonary response to bleomycin injury includes elevations in TSLP and IL-9, which in turn partially ablate the antifibrotic effect of ST2 deficiency. These findings suggest that therapeutic targeting of ST2 alone may represent a suboptimal therapeutic strategy. Instead, dual targeting of ST2 and TSLP may be warranted. Furthermore, the study results support our earlier notion that IL-33 is an important profibrotic mediator acting, at least in part, in a receptor-independent fashion [8,9,10,11,20].

The initial cytokine screening experiments in this study approached the mechanism of antifibrotic resistance to ST2 deficiency in an agnostic fashion. Such strategy allowed for unbiased data collection, serendipitously revealing that in two separate animal models and utilizing two distinct cytokine screening technologies, pulmonary levels of IL-9 were elevated in response to injury independently of the presence or absence of ST2 (Supplementary Figures S1 and S2 and Datasets S1–S4). Subsequent experiments with ELISA-based measurements confirmed that in double-hit and single-hit models, elevations in both pulmonary IL-9 and collagen were resistant to ST2 deficiency (Figure 1 and Figure 2). In fact, genetic deficiency of ST2 alone in ST2KO mice or in combination with genetic deficiency of IL-33 in DKO mice caused a more pronounced elevation in pulmonary IL-9 in response to lung injury than that observed in wild-type mice (Figure 1A). Similarly, pulmonary levels of IL-9 were enhanced by ST2 deficiency in ST2KO mice (Figure 2A). The association between ST2 deficiency-resistant elevations in both IL-9 and collagen might be indicative of a causal link in which the ST2 deficiency-driven elevation in IL-9 compensated for the lack of the profibrotic effect of ST2 through the profibrotic action of IL-9. In other words, IL-9 elevation might represent a redundant compensatory profibrotic mechanism in the absence of ST2. This possibility was experimentally addressed by blocking IL-9 or its upstream regulator TSLP with neutralizing antibodies.

Consistent with the previous reports of others [40,49], IL-9 blockade partially inhibited collagen accumulation in the lungs of WT mice. However, the same treatment failed to attenuate the collagen levels in ST2KO or DKO mice (Figure 2B,C). There were at least two possible interpretations for the latter observation. One, it was possible that in ST2-deficient mice, one or more profibrotic factors other than IL-9 drive pulmonary fibrosis. Two, the possibility could not be excluded that the degree of elevation of IL-9 in ST2-deficient mice is so substantial that the blocking antibody was unable to notably neutralize its profibrotic action. Indeed, in the context of cytokine-driven regulation, local concentration of a cytokine, in this case IL-9, might be particularly high in the local microenvironment of two interacting cells. Such high local concentrations would not be necessarily reflected in the overall levels of the cytokine in the tissue homogenate. Furthermore, the intercellular spaces in the tissues may not always be readily accessible by an antibody. We therefore considered the possibility that the elevated IL-9 expression, which is known to be driven by TSLP [35,36] could be more efficiently blocked upstream of this pathway, by neutralizing TSLP.

Indeed, the levels of pulmonary TSLP were not abrogated by ST2 deficiency. Comparison of the kinetic profiles indicated an earlier elevation and earlier decline in pulmonary levels of TSLP compared to IL-9 levels (Figure 3A, compare to Figure 2A), consistent with the notion of TSLP being an upstream regulator of IL-9 levels. The observed effects of TSLP blockade on pulmonary IL-9 levels, collagen levels, and total body weight (as an indication of the overall disease severity) were particularly important for three reasons. First, these observations supported the conceptual notion that TSLP acts upstream of both IL-9 elevation and collagen production following bleomycin injury. Second, the combined effects of both ST2 deficiency and TSLP blockade exceeded each of their independent effects. Especially notable, the combination of ST2 deficiency and TSLP blockade attenuated collagen levels nearly completely, to the basal levels seen in saline-challenged mice (Figure 3C). This striking effect was paralleled by the pronounced decrease in pulmonary IL-9 (Figure 3B) as well as restoration of the total body weight indicative of alleviation of the overall disease severity (Figure 3D). Finally, a practical implication of the second observation is that therapeutic targeting of either ST2 alone or IL-9 alone in human patients with pulmonary fibrosis may be less efficient than simultaneous targeting of these two factors, e.g., with a bispecific neutralizing antibody.

That CD4+ T cells were the main producers of IL-9 (Figure 4) is consistent with a long-standing notion of T cells being important mechanistic contributors to pulmonary fibrosis [reviewed in [1,2,3,4,5,6]]. Despite voluminous and diverse evidence for such contributions accumulated over the past several decades, there have been limited, if any, attempts to target T cells therapeutically in human patients. This is perhaps due to diverse, both pro- and antifibrotic roles that T cells have been shown to play in the lungs. An argument could be made that instead of indiscriminate targeting of all, pro- and anti-fibrotic, T cells, their specific subsets or their fibrosis-regulating mediators should be targeted. Our data suggest that targeting Th9 cells and, perhaps more promisingly, their upstream regulator, TSLP, may be therapeutically beneficial, especially if such targeting is combined with simultaneous targeting of ST2. Although, as the Introduction section above reflects, the role that IL-9 plays in pulmonary fibrosis appears to be controversial, its profibrotic role comes to the forefront based on relevant specifics of previous studies combined with our current findings. Many of the previous studies have been performed in mice that have been genetically engineered to overexpress IL-9. Super-physiological overexpression of IL-9 induce effects that are different from those occurring due to natural disease-associated changes in IL-9 expression levels. Truly quantitative data on pulmonary levels of IL-9 protein or Th9 cell numbers are limited to only a few publications that report results in humans or WT murine disease models [40,49]. Both articles provide convincing evidence for the profibrotic role of IL-9 in the lungs, and our findings presented here add to this overall conclusion.

There are certain limitations to this study. The effects of the depletion of the IL- 33/S2 axis components were studied in germline-deficient and not in inducible deficiency, tissue- or cell type-selective deficiency, or pharmacologically inhibited models. The possibility cannot be excluded that the ubiquitous constitutive deletion of ST2 and IL-33 may have induced ontogenetic adaptations of the regulatory pathways such that do not occur in more physiologically- or disease-relevant situations. Furthermore, the complete and constitutive genetic abrogation of this important regulatory pathway bears somewhat limited relevance to the typically transient and often incomplete inhibition of therapeutic targets in clinical settings. It is possible that pharmacological blockade of ST2 or IL-33 may elicit effects that are distinct from those of germline deficiency of these molecules. In fact, it is difficult to predict whether prolonged targeting of ST2 in human patients would bring forward its direct moderate antifibrotic effect or, alternatively, act oppositely by emphasizing the profibrotic contribution from the activated TSLP–IL-9 axis. Moreover, the approach utilized in this study did not account for the temporal aspect of disease progression. Therapeutic inhibition of the same molecular target at different stages of disease trajectory may induce distinct effects on pathophysiological mechanisms. A more general and more common limitation of experimental studies in animal models is that they do not always translate into similar responses in humans. Molecular mechanism of the process leading to the observed ST2 deficiency-induced elevation in pulmonary IL-9 have not been addressed and will be the subject of future explorations. Despite these limitations, new and potentially impactful knowledge has been gained from this study as discussed above.

In summary, this study explained the overall mechanism which drives the incomplete attenuation of pulmonary fibrotic response in the absence of ST2. The data indicate that while moderately attenuating the fibrotic response, ST2 deficiency simultaneously activates the profibrotic TSLP–IL-9 axis. The upstream blockade of this axis at the level of TSLP attenuates the induction of IL-9 and ensures a nearly complete abrogation of collagen accumulation in response to profibrotic injury. These findings have important implications, both conceptual and practical. Conceptually, this report supports and expands our long-standing notion that pulmonary fibrosis is not necessarily predominantly driven by the conventional IL-33/ST2 cytokine/receptor axis [8,9,10,11]. Full-length IL-33 contributes to pulmonary fibrosis in a receptor-independent manner, [8,9,10,11], and based on the data presented in this report, ST2 regulates the fibrotic response independently of the presence or absence of IL-33. Perhaps the regulation of fibrosis by IL-33 and ST2 should be considered beyond the traditional focus on the IL-33/ST2 axis, to include their independent fibrosis-regulating contributions. It is unknown whether the same notion applies across fibrotic situations in organs and tissues other than the lung. This possibility remains to be experimentally explored in future studies. In terms of potential targeting of ST2 in patients with pulmonary fibrosis, our findings suggest that the sole therapeutic targeting of ST2 is unlikely to be highly efficacious. A bispecific therapy simultaneously targeting ST2 and TSLP should be considered, or, alternatively, efforts should be focused away from targeting the IL-33/ST2 axis to other promising pathways, such as the ST2-independent fibrotic mechanism driven by the full-length IL-33 precursor [7,8,9,10,11].

## 4. Materials and Methods

### 4.1. Experimental Animals

The animal studies were performed in accordance with a research protocol reviewed and approved by the University of Maryland Institutional Animal Care and Use Committee. Sex as a biological variable was addressed by including approximately equal numbers of male and female mice in each experimental group in each experiment. Animals were maintained in sterile microisolator cages with sterile rodent feed and water. Daily maintenance of mice was performed at the Baltimore VA Medical Center Research Animal Facility, which is approved by the Association for Assessment and Accreditation of Laboratory Animal Care. All mouse strains were on the C57BL/6 background. Wild-type (WT) C57BL/6 mice aged 10–12 weeks (The Jackson Laboratory, Bar Harbor, ME, USA). Homozygously germline-deleted, i.e., gene knockout (KO), animals were also used, including ST2^−/−^ (ST2KO), and IL33^−/−^ST2^−/−^ double knockout (DKO) mice that were previously described [8,9,10,11]. Both KO strains were on the C57BL/6 background.

### 4.2. Bleomycin Injury Models and Experimental Treatments

Acute intratracheal bleomycin injury model was used as previously described [7,9,10]. Briefly, a single dose of 0.075 U of bleomycin (Sigma-Aldrich, St. Louis, MO, USA) diluted in 50 μL of sterile phosphate-buffered saline was delivered intratracheally (IT) to mice on day 0. For the IT instillation, a minor anterior midline neck incision was made to reveal the trachea. A Micro Sprayer (Penn-Century, Wyndmoor, Philadelphia, PA, USA) was inserted intratracheally and bleomycin or sterile PBS instilled. On the days indicated for specific experiments in the Results section, typically days 7, 14, or 21 after the day of bleomycin injection (day 0), mice were euthanized by CO_2_ asphyxiation, followed by cervical dislocation.

In some single-hit bleomycin injury experiments, animals received neutralizing anti-IL-9 or anti-TSLP antibodies. These in vivo blocking antibodies were from BioXCell (Lebanon, NH, USA), including anti-mouse IL-9 mAb (cat. #BE0181) and its isotype control mouse IgG2a (cat. #BE0085), as well as anti-mouse TSLP (cat. #BE0379) and its rat IgG2a isotype control (cat. #BE0089). The anti-IL-9 antibody or its isotype control were used at 100 µg per mouse per injection. The anti-TSLP antibody or its isotype control were used at 20 µg per mouse per injection. Each of these blocking antibodies or isotype controls were administered intraperitoneally on days −2, −1, 0 (the day of bleomycin challenge), 1, 3, 5, 7, 9, 11, 13, 15, and 18, and mice were euthanized on day 21.

A double-hit model was also utilized based on combining pulmonary overexpression of either FLIL33 or MIL33 with subsequent acute bleomycin injury. Replication-deficient recombinant adenovirus (AdV)-based overexpression in vivo was used for the “first hit.” The AdV constructs were prepared and used as previously described [8,9,10,11]. The AdV-FLIL33 or AdV-MIL33 vectors encoding mouse FLIL33 or MIL33, respectively, or the AdV-NULL vector not encoding a cytokine were instilled IT on day 0. This was followed by the IT bleomycin challenge 7 days later, with subsequent analyses of pulmonary changes on day 21 after AdV instillation (corresponding to day 14 after bleomycin instillation), as previously described [8,9,10].

In both models, the lungs were extracted immediately postmortem and analyzed as described below.

### 4.3. Experimental Readouts

In some experiments, lung homogenates were prepared by snap-freezing the lungs and crushing them under liquid nitrogen, followed by thawing in 0.5 mL of PBS containing a protease inhibitor cocktail (Sigma, St. Louis, MO, USA) with subsequent homogenization in a glass homogenizer. For measurement of total lung collagen, the homogenates were analyzed using QuickZyme (Leiden, The Netherlands) approach based on hydroxyproline content. For measurements of cytokine levels, the solid content of the homogenates was removed by centrifugation. The supernatant was diluted with the ELISA sample buffer and used for the measurements of cytokine levels by either Olink (Target 48 Cytokine Mouse panel, Uppsala, Sweden) or multiplex (Luminex, Austin, TX, USA) approaches, or by ELISA for IL-9 or TSLP (both from R&D Systems, Minneapolis, MN, USA).

In some experiments, mouse lungs were dissociated by incubation with occasional mixing for 40 min at 37 °C in RPMI medium containing 0.2 mg/mL of type 1 collagenase (Sigma) and 0.1 mg/mL DNase (Roche, Basel, Switzerland), followed by filtering through a 70 µm nylon cell strainer with subsequent centrifugation at 1200 rpm for 8 min at 4 °C. Thus prepared unseparated lung cells were used for flowcytometric analyses and well as for purification of pulmonary T cells for subsequent immunocytochemical analysis.

Flowcytometric analyses of unseparated pulmonary cells were performed on a Cytek (Fremont, CA, USA) Aurora spectral flow cytometer, and data were analyzed using FlowJo software version 10 (TreeStar, San Carlos, CA, USA). Cells were stained with fluorochrome-conjugated antibodies at saturating concentrations. All antibodies were purchased from BD Biosciences (San Jose, CA, USA), BioLegend (San Diego, CA, USA), or eBioscience (Santa Clara, CA, USA).The following antibodies were used: CD45 (BUV395, clone 30-F11), CD4 (BUV496, clone GK1.5), Lineage cocktail (eFluor™ 450; anti-CD3 [17A2], anti-CD45R/B220 [RA3-6B2], anti-CD11b [M1/70], anti-TER-119 [TER-119]), and anti-Ly-6G/Gr-1 [RB6-8C5]), CD8 (FITC, clone 53-6.7), CD3 (PE, clone 145-2C11), ST2 (PE-Cy7, clone DJ8), IL-9 (APC, clone RM9A4), and CD90.2 (AF700, clone 30-H12). Dead cells were excluded using Live/Dead Yellow viability dye. For intracellular IL-9 staining, cells were treated with GolgiStop (BD Biosciences, Franklin Lakes, NJ, USA), then fixed and permeabilized using the eBioscience intracellular fixation/permeabilization buffer set.

For purification of pulmonary T cells, the Dynabeads Untouched™ Mouse T Cells Kit (Invitrogen, Carlsbad, CA, USA) was used per manufacturer’s specifications. Cytospin slides of purified permeabilized lung T cells were stained for IL-9 using an Abcam (Waltham, MA, USA) rabbit monoclonal antibody (cat. #ab227037) in 1:500 dilution. Nuclei were stained with DAPI. The slides were viewed on a Zeiss LSM700 confocal microscope (Oberkochen, Germany), using a 63× NA 1.4 oil immersion objective. The images were analyzed using the Zeiss ZEN version 3.5 software package. The gain and the offset setting for both the DAPI and FITC channels on the microscope were adjusted to prevent saturation of the signal in the cells that were stained with IL-9 and DAPI.

### 4.4. Statistical Analyses

Experimental data were expressed as individual and mean values. One-way ANOVA across experimental groups followed by post hoc tests for pairwise comparisons was performed utilizing Jamovi software version 2.6 [52].

## Data Availability

The original contributions presented in this study are included in the article/Supplementary Materials. Further inquiries can be directed to the corresponding authors.

## Supplementary Materials

The following supporting information can be downloaded at: https://www.mdpi.com/article/10.3390/ijms262411787/s1.

## Abbreviations

The following abbreviations are used in this manuscript:

DKO	double knockout, in this case IL33KOST2KO
IL	interleukin
ILC	innate lymphoid cell
ILD	interstitial lung disease
IPF	idiopathic pulmonary fibrosis
KO	knockout (gene deletion)
SSc	systemic sclerosis
ST2	serum stimulation-2, same as IL1RL1 or T1 or IL-33 receptor
TSLP	thymic stromal lymphopoietin
