# Supplementary material for: Activation of TSLP–IL-9 Axis Hinders the Antifibrotic Effect of ST2 Deficiency in Pulmonary Fibrosis
Source: Int J Mol Sci. 2025 Dec 5;26(24):11787. doi: 10.3390/ijms262411787 (PMC12732439; doi:10.3390/ijms262411787)
Supplement: Supplementary file 1 [file ijms-26-11787-s001.zip › supplementary_figures.pdf]

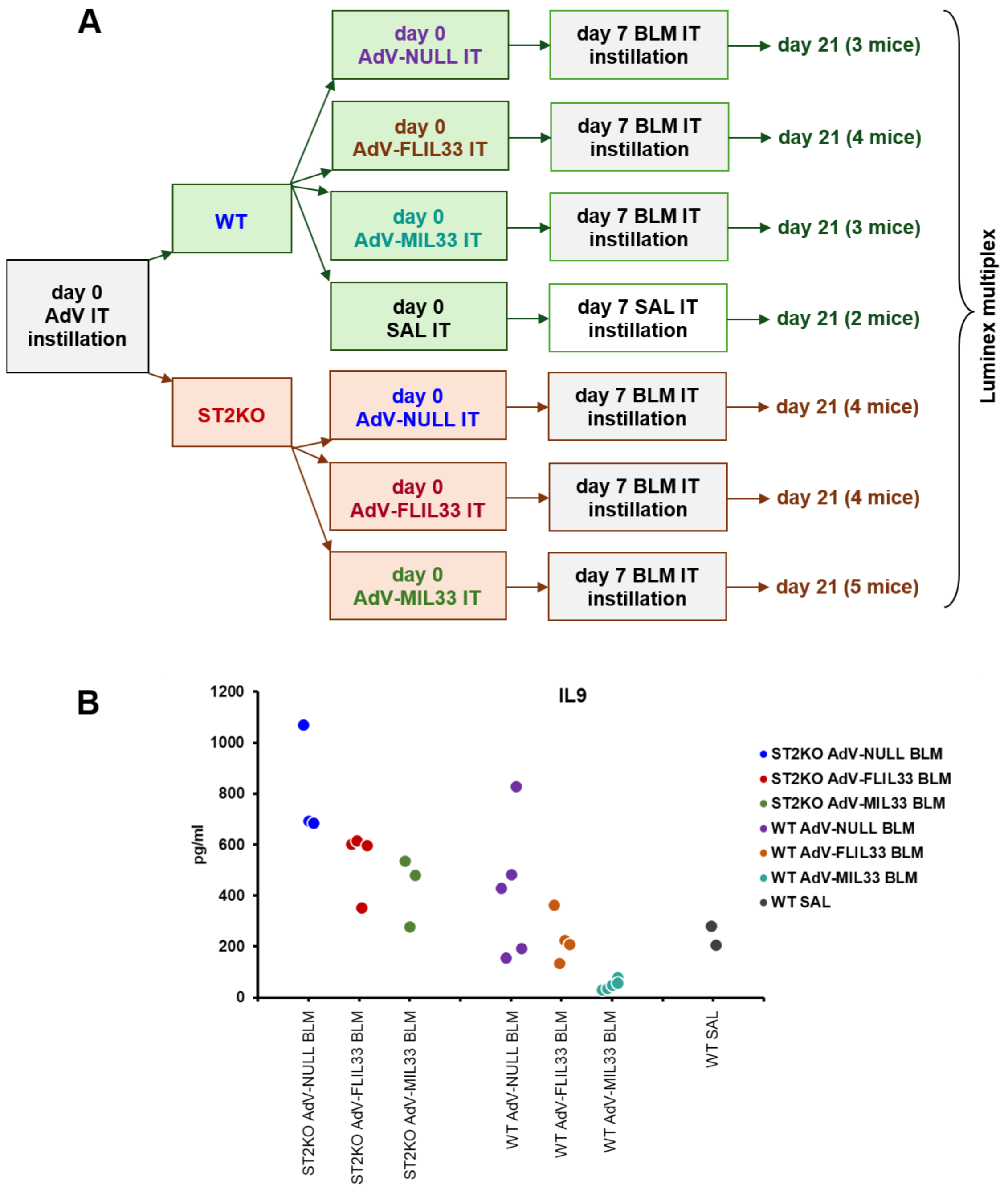

Supplementary Figure S1. Screening of pulmonary cytokines in the double-hit model of pulmonary fibrosis. **A.** Schematic outline of the pulmonary cytokine level screening experiment utilizing Luminex multiplex technology. On day 0, ST2KO and WT mice received IT instillations of replication-deficient recombinant adenoviral (AdV) constructs encoding full-length IL-33 (AdV-FLIL33), mature IL-33 (AdV-MIL33), or not encoding a cytokine (AdV-NULL). Two WT mice received IT saline (SAL) as a control. On day 7, all animals, except for the two mice in the saline control group, were challenged with IT bleomycin (BLM), whereas the two control mice again received IT saline. The indicated numbers of mice were euthanized on day 21, their lungs collected immediately postmortem and homogenized, and the homogenates assayed for elected cytokines using Luminex multiplex approach. **B.** Pulmonary levels of IL-9 in lung homogenates based on Luminex multiplex screening. All homogenates were similarly diluted for testing; the measured values were plotted without adjustment for the dilution factor.

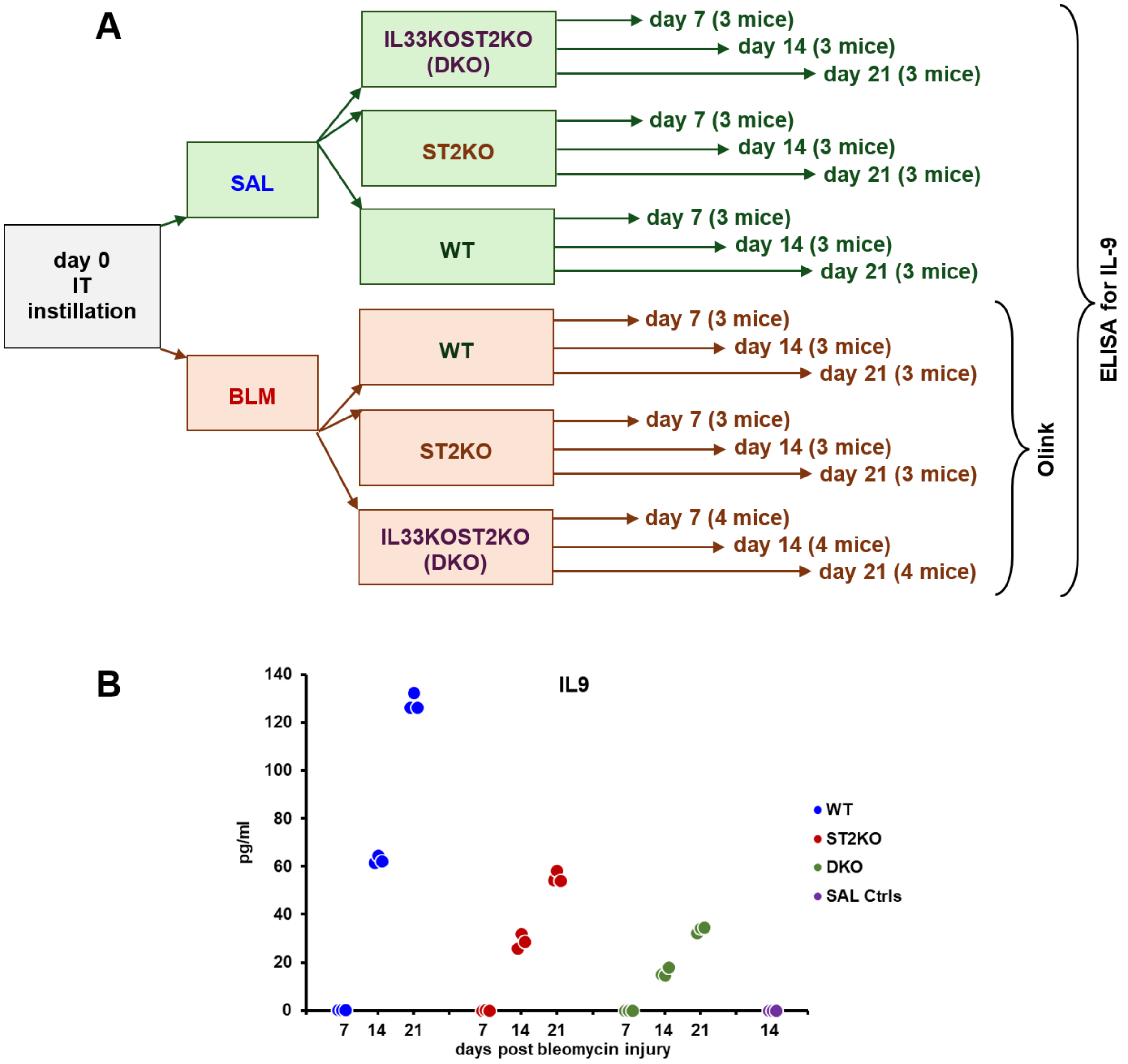

Supplementary Figure S2. Screening of pulmonary cytokines in the single-hit model of pulmonary fibrosis. **A.** Schematic outline of the pulmonary cytokine level screening experiment utilizing Olink technology. On day 0, ST2KO, DKO, and WT mice received IT instillations of bleomycin (BLM) or saline (SAL) control. On days 7, 14, and 21, the indicated numbers of mice in each group were euthanized, their lungs collected immediately postmortem and homogenized, and the homogenates assayed using the Olink Target 48 Mouse Cytokine panel as well as by ELISA for murine IL-9. **B.** Pulmonary levels of IL-9 in lung homogenates based on Olink screening. All homogenates were similarly diluted for testing; the measured values were plotted without adjustment for the dilution factor. The majority of the tested samples were derived on days 7, 14, and 21 post bleomycin challenge from WT, ST2KO, and DKO mice, as indicated. In the “SAL Ctrls” group, one of each ST2KO, DKO, and WT saline-challenged, day 14, controls were included to show basal expression of each cytokine across the four strains.
